# Self-harm in a primary care cohort of older people: incidence, clinical management, and risk of suicide and other causes of death

**DOI:** 10.1016/S2215-0366(18)30348-1

**Published:** 2018-11

**Authors:** Catharine Morgan, Roger T Webb, Matthew J Carr, Evangelos Kontopantelis, Carolyn A Chew-Graham, Nav Kapur, Darren M Ashcroft

**Affiliations:** a Centre for Mental Health and Safety, Faculty of Biology, Medicine and Health, NIHR Greater Manchester Patient Safety Translational Research Centre, Manchester Academic Health Science Centre, The University of Manchester, Manchester, UK; b National Institute for Health Research (NIHR) School for Primary Care Research, Division of Informatics, Imaging and Data Sciences, Faculty of Biology, Medicine and Health, NIHR Greater Manchester Patient Safety Translational Research Centre, Manchester Academic Health Science Centre, The University of Manchester, Manchester, UK; c Centre for Suicide Prevention, Faculty of Biology, Medicine and Health, NIHR Greater Manchester Patient Safety Translational Research Centre, Manchester Academic Health Science Centre, The University of Manchester, Manchester, UK; d Centre for Pharmacoepidemiology and Drug Safety, Faculty of Biology, Medicine and Health, NIHR Greater Manchester Patient Safety Translational Research Centre, Manchester Academic Health Science Centre, The University of Manchester, Manchester, UK; e Research Institute, Primary Care and Health Sciences, West Midlands, Collaboration for Leadership in Applied Health Research and Care, Keele University, Staffordshire, UK; f Greater Manchester Mental Health NHS Foundation Trust, Prestwich Hospital, Prestwich, Manchester, UK

## Abstract

**Background:**

Self-harm is a major risk factor for suicide, with older adults (older than 65 years) having reportedly greater suicidal intent than any other age group. With the aging population rising and paucity of research focus in this age group, the extent of the problem of self-harm needs to be established. In a primary care cohort of older adults we aimed to investigate the incidence of self-harm, subsequent clinical management, prevalence of mental and physical diagnoses, and unnatural-cause mortality risk, including suicide.

**Methods:**

The UK Clinical Practice Research Datalink contains anonymised patient records from general practice that routinely capture clinical information pertaining to both primary and secondary care services. We identified 4124 adults aged 65 years and older with a self-harm episode ascertained from Read codes recorded during 2001–14. We calculated standardised incidence and in 2854 adults with at least 12 months follow-up examined the frequency of psychiatric referrals and prescription of psychotropic medication after self-harm. We estimated prevalence of mental and physical illness diagnoses before and after self-harm and, using Cox regression in a matched cohort, we examined cause-specific mortality risks.

**Findings:**

Overall incidence of self-harm in older adults aged 65 years and older was 4·1 per 10 000 person-years with stable gender-specific rates observed over the 13-year period. After self-harm, 335 (11·7%) of 2854 adults were referred to mental health services, 1692 (59·3%) were prescribed an antidepressant, and 336 (11·8%) were prescribed a tricyclic antidepressant (TCA). Having a diagnosed previous mental illness was twice as prevalent in the self-harm cohort as in the comparison cohort (prevalence ratio 2·10 [95% CI 2·03–2·17]) and with a previous physical health condition prevalence was 20% higher in the self-harm cohort compared to the comparison cohort (1·20 [1·17–1·23]). Adults from the self-harm cohort (n=2454) died from unnatural causes an estimated 20 times more frequently than the comparison cohort (n=48 921) during the first year. A markedly elevated risk of suicide (hazard ratio 145·4 [95% CI 53·9–392·3]) was observed in the self-harm cohort.

**Interpretation:**

Within primary care, we have identified a group of older adults at high risk from unnatural death, particularly within the first year of self-harm. We have highlighted a high frequency of prescription of TCAs, known to be potentially fatally toxic in overdose. We emphasise the need for early intervention, careful alternative prescribing, and increased support when older adults consult after an episode of self-harm and with other health conditions.

**Funding:**

National Institute for Health Research Greater Manchester Patient Safety Translational Research Centre.

## Introduction

Suicide is a major public health issue worldwide. With consideration of socioeconomic differences between regions, suicide risk rises with increasing age.^1^, ^2^ Data routinely reported from the Office for National Statistics (ONS) in England and Wales indicate that suicide rates in older women have increased in the past 5 years, converging toward those of younger women of working age. In men aged 60 years and older, suicide rates have increased from 12·3 per 100 000 population in 2012 to 14·8 per 100 000 in 2015, which is higher than rates for male adolescents and younger male adults (10–29 years) at 10·6 per 100 000 in 2015.^3^

Non-fatal self-harm is the strongest risk factor for subsequent suicide.^4^, ^5^ Unlike other age groups, older people who self-harm have an increased suicidal intent;^6^ thus, although repetition rates are low compared with middle-aged adults, self-harm is more often fatal in older adults.^5^, ^6^, ^7^ In a UK multicentre study^4^ of people with self-harm episodes presenting in hospital, suicide risk was 67 times higher among people aged 60 years and older who had harmed themselves versus their peers who had not, and was three times higher than younger individuals aged 20–59 years who had harmed themselves. It is, therefore, surprising that self-harm among older people has received little attention compared with other age groups. The scarce published evidence on self-harm in people of an older age consists mostly of investigations based on hospital emergency department attendance,^4^, ^5^, ^6^, ^8^, ^9^, ^10^ mortality statistics, or register-based studies.^8^, ^10^, ^11^ Most report on risk factors for self-harm repetition or suicide^4^, ^9^, ^10^, ^11^ or describe incidence, self-harm method, and suicide as a primary outcome.^5^, ^6^, ^8^

**Research in context**

**Evidence before this study**

We searched Embase and PubMed with initial title terms related to self-harm (“self-harm”, “self-injur*” “self-mutilation”, “self-poisoning”, “self-destructive”, “suicide”) and an older population (“aged”, “ageing”, “aging”, “elderly”, “old”, “older”). We included further combination terms related to epidemiology of self-harm, including “mortality”, “epidemiol*”, “prevalence”, “incidence”. We checked publications covering all ages for adult populations to ensure older age bands were included in results sections. Further combination searches for clinical management, including referrals and treatment prescribing, and mental and physical diagnoses, were also included. We searched for articles published from Jan 1, 2000, to Dec 31, 2017, and set no restriction on language, method, or quality of publication. We also checked citations of relevant publications. No publications were found on self-harm specific to older age populations at primary care level. Most studies relating to self-harm among adults of an older age were based on hospital emergency department attendance, mortality statistics, or register-based studies of adult populations of all ages. These reported on risk factors for self-harm repetition or suicide or described incidence, self-harm method, and suicide as a primary outcome. Clinical management in older adults after self-harm was rarely reported. One multicentre, hospital-based study in England reported self-harm across all adult age ranges and provided some data on referrals after hospital admission. We did not identify any publications on prescribing patterns in older adults after self-harm. One US study, investigating specific self-poisonings, detailed medication use at the time of self-harm. Reports on mental and physical illness diagnoses specific to self-harm populations were scarce. Most presented illness diagnoses as risk factors to suicide outcome and were from small psychological autopsy studies. Mortality risk literature was based only in hospital settings.

**Added value of this study**

This study is a novel primary-care-based study with linkage to hospital and mortality records to establish the largest primary care dataset of self-harm in older adults of its kind. We have identified a group of older adults who registered with a general practice as being at high risk of premature unnatural death, particularly within the first year of a self-harm episode. Further, we have identified low referral rates contrary to National Institute for Health and Care Excellence (NICE) guidelines, which suggest that older adults should be seen by a mental health professional after an episode of self-harm. Additionally, we have identified a high rate of prescription of tricyclic antidepressants (TCAs); NICE guidelines clearly recommend their avoidance after self-harm. We have also shown an increased prevalence of psychiatric and physical diagnoses before self-harm, previously shown to be important risk factors for self-harm.

**Implications of all the available evidence:**

Our findings suggest that health-care professionals in primary care have the opportunity to consider self-harm risk when older adults consult with complex mental or physical health-care needs. Additionally, after self-harm in an older adult, improvement of referral rates and consideration of possible alternative medication, with particular avoidance of TCAs, might reduce the risk of escalating self-harm behaviour and associated mortality risk.

A 3% annual increase has been seen in the proportion of adults aged 60 years and older in the global population.^12^ In the UK population, the projected number of individuals aged 65 years and older is set to rise to 25% of the UK population by 2046.^13^ Older adults often face different psychosocial stressors to young or middle-aged adults. Decline in functional ability through multiple comorbid conditions,^9^, ^11^, ^14^ experience of bereavement, or potential social isolation^5^ are all associated with self-harm among older adults as a behavioural response to psychological distress. Therefore, the consideration of mental well-being, as endorsed by WHO to include all age groups across the life course^15^ and to align health-care services to meet both physical and mental health needs in older-aged populations, is important and timely.^16^

In England, the National Institute for Health and Care Excellence (NICE) clinical guidance recommends that older adults are assessed by an old-age psychiatrist or mental health specialist after a self-harm episode because of raised levels of suicidal intent at older age.^17^ However, previous studies of self-harm in older people often do not have detail on specific clinical management after the index episode. A multicentre, hospital-based study^4^ in England reported that 36% of adults aged 60 years and older were referred back to their general practitioner (GP) after self-harm without onward referral to specialist services, contrary to the NICE guidelines. Clearly, due to this poor level of compliance with national guidelines, the clinical management of older adults after self-harm needs to be explored to establish where improvements in clinical care could be made for effective suicide prevention initiatives to be implemented. Furthermore, no published studies have investigated self-harm among older adults registered with a GP. Examination of only episodes presenting in hospital might underestimate self-harm incidence among older adults. Using electronic patient records, we aimed to investigate self-harm incidence, its subsequent clinical management, and unnatural-cause mortality risk in a large cohort of older adults registered with a GP.

## Methods

### Data sources

Electronic primary care patient records, containing routinely collected information on symptoms, diagnoses, prescribed medication, and referrals to secondary care services, were extracted from the Clinical Practice Research Datalink (CPRD). This source contains over 4·4 million active patient records from 674 registered general practices, covering 6·9% of the UK population. It is broadly representative of the national population in terms of age, gender, and ethnicity.^18^ A subset of CPRD-registered practices located in England participate in the CPRD linkage scheme (approximately 60% of all UK CPRD practices), which enables routine linkage to ONS death registrations, Hospital Episode Statistics (HES) diagnostic coding for inpatient episodes (with the ICD-10), and the 2010 Index of Multiple Deprivation (IMD)—an ecological socioeconomic indicator.

### Delineation of the study cohort

The study cohort consisted of adults aged 65 years and older with a self-harm episode recorded during calendar years from 2001 to 2014. Self-harm episodes were identified by Read codes from patients' electronic primary care health records, which capture health-care interaction from multiple sources, including information captured during a GP consultation and notification of an episode to the patient's GP after hospital attendance. Self-harm was defined, as in NICE Clinical Guideline CG16,^17^ as “any act of self-poisoning or self-injury, irrespective of motivation”. In some studies, self-harm classifications of non-suicidal self-injury or suicide attempt are applied, but these are reliant on establishing suicidal intent, a determination that cannot be made on the basis of the clinical Read codes^19^ recorded in the CPRD. Self-harm episodes in patients' primary care records were therefore identified from a spectrum of candidate Read codes from milder forms of non-suicidal behaviour to near-fatal attempted suicide, with each code subject to rigorous clinical review (CAC-G, NK).^20^ The patient inclusion criteria for each analytical phase of the study are outlined in detail in the appendix.

### Multiphase study designs and statistical analyses

In the first analytical phase, we calculated annual incidence from the number of older adults with a record of self-harm during each calendar year in relation to the total number of older adults registered with a GP who were at risk during the same year. Stratified on age band, IMD, and region of residence, we applied strata-specific rates to calculate directly standardised incidence of self-harm as the outcome. The IMD is an ecological deprivation measure derived as a composite of several socioeconomic indicators for small areas based on practice location or patient's residential postcode.^21^, ^22^ IMD is ranked within each UK country (England, Scotland, Wales, and Northern Ireland) and is then placed in quintiles from least to most deprived areas. We categorised age in three age bands: 65–74, 75–84, and 85+ years.

In the second analytical phase, we assessed clinical management as an outcome according to referrals to specialist mental health services and prescription of psychotropic medication by a GP during the year after the index self-harm episode. We captured psychiatric referrals using the Family Health Services Authority Psychiatry code, National Health Service specialty fields, and further Read codes identified as indicating a psychiatric referral. Psychotropic medication included prescriptions issued for antidepressants, antipsychotics, and hypnotics or anxiolytics. We stratified clinical management variables by gender, age group, and IMD quintile on the basis of general practice location.

For the third analytical phase, we implemented a matched cohort study design to describe mental and physical illness prevalence as an outcome in both a self-harm and comparison cohort and to investigate mortality risk as an outcome after self-harm. We restricted the cohort to patients registered and up-to-standard for research purposes at general practices for at least a year before cohort entry, within England, and participating in the CPRD-HES-ONS linkage scheme. We included incident self-harm episodes recorded in the CPRD between Jan 1, 2001, and March 31, 2014, in which no previous self-harm episode was identified from both the primary care record and via linkage to HES records from ICD-10 codes X60–X84.9. Each incident episode was matched by age, gender, and registered practice with up to 20 comparison individuals with no record of self-harm on the index self-harm date of its corresponding matched case. We opted to sample this many patients in the comparison cohort to maximise statistical power and precision for examination of unnatural death as a rare outcome.

We identified psychiatric and physical illness diagnoses recorded before index date entry and separately identified new diagnoses after the index date from Read and ICD-10 codes recorded in CPRD or linked HES records in both the self-harm and comparison cohorts. The psychiatric diagnostic categories included depression, anxiety disorders, personality disorders, bipolar disorder, schizophrenia-spectrum disorders, and dementia. Physical health diagnoses, based on the Charlson comorbidity index, included cancer, cerebrovascular disease, chronic pulmonary disease, congestive heart disease, diabetes with and without complications, hemiplegia, metastatic tumour, mild and moderate liver disease, myocardial infarction, peptic ulcer disease, peripheral vascular disease, renal disease, and rheumatological disease. We generated dichotomous indicator variables for any psychiatric or physical illness. Additionally, we calculated the Charlson comorbidity index scores^23^ at cohort entry.

We defined classification of cause-specific mortality using the underlying cause of death code from ICD-10 and included all natural deaths (all codes, excluding V01–Y98), all unnatural deaths (V01–Y98), and suicides (including open verdicts X60–X84 and Y10–Y34, excluding Y33.9, Y87.0, and Y87.2). To estimate the risk of all-cause and cause-specific mortality, we used Cox regression modelling, producing both unadjusted hazard ratios [HRs] and HRs adjusted by IMD quintile based on a patient's postcode. Adjustments by mental illness diagnoses were not appropriate in this instance because these were suspected to be on the causal pathway. We did, however, report prevalence of diagnosis for serious mental illness before and after the index date. The index date of the self-harm cohort was defined as the occurrence of the first ever recorded self-harm event. Follow up started from the index date in both the self-harm cohort members and their matched comparators. Records were right-censored at the date of the first occurrence of either the end of the follow-up period (March 31, 2014), the practice last data collection date, date of transfer out of the CPRD (ie, patient registered with a new practice or practice that left the CPRD), or death. We evaluated the proportional hazards assumption formally using Schöenfeld's residuals.^24^ Additionally, we estimated the cumulative incidence percentage values for all-cause mortality and specific causes of death, adjusted for competing risk of dying from other causes.^25^

All code lists applied in this study are published online and we used Stata/SE 14.2 for all analyses.

### Role of the funding source

The funders of the study had no role in study design, data collection, analysis, or interpretation. The corresponding author had full access to all the study data and final responsibility for publication submission.

## Results

During the 2001–14 observation period, 4124 adults aged 65 years and older were identified as having an incident self-harm episode. Of these, 3327 (80·7%) involved ingestion of drugs, 234 (5·7%) involved self-cutting, a further 118 (2·9%) were by some other means, and 435 (10·6%) had no method specified. The overall incidence was 4·1 per 10 000 person-years and stable gender-specific incidences were observed across the entire 13-year period (appendix). Across the whole observation period, similar standardised incidences for self-harm were observed in men and women (table 1; women to men rate ratio 1·03 [95% CI 0·97–1·10]). Compared with the reference group of people aged 65–74 years, incidence of self-harm increased by 31% among 75–84 year olds (rate ratio 1·31 [95% CI 1·23–1·41]) and by 76% among patients 85 years and older (1·76 [1·62–1·91]). For practice-level deprivation, the rate ratios were significantly raised for the middle and most deprived IMD quintiles (ie, for quintile 3, rate ratio 1·14 [95% CI 1·04–1·26]) and for quintile 5, 1·16 [1·05–1·29]; table 1).

**Table 1:** Standardised incidence and rate ratios stratified by sex, age, and socioeconomic deprivation

		**Number of people who self-harmed**	Adjusted incidence*(95% CI) per 10 000 person-years	**Rate ratio (95% CI)**
All people		4124	4·08 (3·95–4·20)	..
Gender				
	Female	2392 (58·00%)	4·03 (3·84–4·22)	1·00 (ref)
	Male	1732 (42·00%)	4·16 (4·00–4·33)	1·03 (0·97–1·10)
Age, years				
	65–74	1722 (41·76%)	3·35 (3·19–3·51)	1·00 (ref)
	75–84	1541 (37·37%)	4·40 (4·18–4·62)	1·31 (1·23–1·41)
	85+	861 (20·88%)	5·90 (5·49–6·31)	1·76 (1·62–1·91)
Index of Multiple Deprivation quintile				
	1 (least)	717 (17·39%)	3·66 (3·32–4·00)	1·00 (ref)
	2	787 (19·08%)	4·03 (3·71–4·34)	1·10 (0·99–1·22)
	3	944 (22·89%)	4·19 (3·89–4·48)	1·14 (1·04–1·26)
	4	845 (20·49%)	3·92 (3·64–4·20)	1·07 (0·97–1·18)
	5 (most)	831 (20·15%)	4·25 (3·88–4·61)	1·16 (1·05–1·29)

Data are n (%) unless otherwise specified. Ref=reference.

* Standardised by sex, age, geographical region, and Index of Multiple Deprivation quintile.

Within 12 months of their initial self-harm episode, 335 (11·7%) of 2854 older adults were referred to mental health services, although more women were referred than men (13·1% *vs* 9·7%; χ^2^=7·84; p=0·005; table 2). Additionally, older adults from general practices in the most deprived localities were 33% less likely to be referred in the first 12 months after an index self-harm episode compared with older adults from practices in the least deprived areas (HR 0·67 [95% CI 0·45–0·99]). In women, 1288 (75·1%) of 1716 were prescribed psychotropic medication compared with 744 (65·4%) of 1138 men (table 2). Psychotropic medication was more frequently prescribed to patients in the youngest age group (65–75 years) versus the oldest age group (85+ years; 73·0% *vs* 66·2%; χ^2^=5·49, 1 df; p=0·01). Women were more often prescribed psychotropic medication than men across all psychotropic medication categories (table 3) and the most frequently prescribed psychotropic medication type was antidepressants (1692 [59·3%] of 2854; table 3). Overall, 336 (11·8%) of 2854 older adults were prescribed a tricyclic antidepressant (TCA) within 12 months of their index self-harm episode. In the year after the index self-harm episode, 412 (14·4%) individuals had a further self-harm episode, of whom 344 (83·5%) had one repeat record and 68 (16·5%) had two or more further episodes.

**Table 2:** Clinical management of adults aged 65 years and older, stratified by demographic subgroups in the year after an index self-harm episode

		**Mental health service referral**	**p value**	Psychotropic medication*	**p value**
All persons		335/2854 (11·7%)		2032 (71·2%)	
Gender					
	Female	225/1716 (13·1%)		1288 (75·1%)	
	Male	110/1138 (9·7%)	χ^2^=7·84†; p=0·005	744 (65·4%)	χ^2^=31·2†; p<0·0001
Age, years					
	65–74	156/1327 (11·8%)		969 (73·0%)	
	75–84	118/1053 (11·2%)		749 (71·1%)	
	85+	61/474 (12·9%)	χ^2^=0·086‡; p=0·77	314 (66·2%)	χ^2^=5·94‡; p=0·01
Index of Multiple Deprivation quintile					
	1 (least)	65/493 (13·2%)		362 (73·4%)	
	2	69/521 (13·2%)		354 (67·9%)	
	3	85/673 (12·6%)		479 (71·2%)	
	4	68/589 (11·5%)		429 (72·8%)	
	5 (most)	48/578 (8·3%)	χ^2^=7·26‡; p=0·007	408 (70·6%)	χ^2^=4·76‡; p=0·31

Data are n/N (%), unless otherwise specified.

* Including antidepressants, antipsychotics, and hypnotics or anxiolytics.

† Pearson χ^2^ test.

‡ Kruskal-Wallis test.

**Table 3:** Prescription of psychotropic medications in the year after index self-harm episode

		**All people (n=2854)**	**Men (n=1138)**	**Women (n=1716)**	**χ^2^ test**
Antidepressants		1692 (59·3%)	621 (54·6%)	1071 (62·4%)	χ^2^=17·44; p<0·0001
	Serotonin reuptake inhibitors	837 (29·3%)	319 (28·0%)	518 (30·2%)	
	Tricyclic antidepressants	336 (11·8%)	108 (9·5%)	228 (13·8%)	
	Other antidepressants	519 (18·2%)	194 (17·0%)	325 (18·9%)	
Antipsychotics		601 (21·1%)	186 (16·3%)	415 (24·2%)	χ^2^=25·30; p<0·0001
	First generation	245 (8·6%)	64 (5·6%)	181 (10·5%)	
	Second generation	356 (12·5%)	122 (10·7%)	234 (13·6%)	
Hypnotics or anxiolytics		1177 (41·2%)	406 (35·7%)	771 (44·9%)	χ^2^=24·18; p<0·0001

Data are n (%), unless otherwise specified.

2454 older adults were in the linked self-harm cohort and 48 921 were in the matched comparison cohort (59·6% women and 40·4% men). A diagnosed previous mental illness was more than twice as prevalent in members of the self-harm cohort than the comparison cohort (prevalence ratio 2·10 [95% CI 2·03–2·17]), as was a subsequent diagnosis of mental illness (2·18 [2·06–2·32]; table 4). Subsequent diagnoses of personality disorder were 14 times more frequent in the self-harm cohort than the comparison cohort and bipolar disorder was 11 times more frequent (table 4). The prevalence of previous diagnosis with a physical health condition was 20% higher in the self-harm cohort than the comparison cohort (prevalence ratio 1·20 [95% CI 1·17–1·23]; appendix). Rising levels of multimorbidity had increased prevalence in the self-harm cohort. The Charlson comorbidity index condition categories of 0, 1, or 2 were more prevalent in the comparison cohort than in the self-harm cohort (prevalence ratio 0·72 [95% CI 0·68–0·76]) and categories 3–5 and greater, which indicated higher multimorbidity levels, were more prevalent in the self-harm cohort (appendix).

**Table 4:** Prevalence of psychiatric illness in the self-harm and comparison cohorts before and subsequent to the index date

	**Self-harm cohort (n=2454)**	**Comparison cohort (n=48 921)**	**Prevalence ratio (95% CI)**
**Depression**			
Before	1139 (46·4%)	8714 (17·8%)	2·61 (2·49–2·73)
Subsequent	374 (15·2%)	1791 (3·7%)	4·16 (3·75–4·62)
**Anxiety disorders**			
Before	857 (34·9%)	8207 (16·8%)	2·08 (1·97–2·20)
Subsequent	177 (7·2%)	1839 (3·8%)	1·92 (1·65–2·23)
**Personality disorders**			
Before	48 (2·0%)	155 (0·3%)	6·17 (4·48–8·51)
Subsequent	12 (0·5%)	17 (<0·1%)	14·07 (6·73–29·43)
**Bipolar disorder**			
Before	78 (3·2%)	216 (0·4%)	7·20 (5·57–9·30)
Subsequent	32 (1·3%)	59 (0·1%)	10·81 (7·04–16·59)
**Schizophrenia-spectrum disorders**			
Before	121 (4·9%)	388 (0·8%)	6·22 (5·09–7·59)
Subsequent	72 (2·9%)	179 (0·4%)	8·02 (6·11–10·51)
**Dementia**			
Before	254 (10·4%)	2047 (4·2%)	2·47 (2·19–2·80)
Subsequent	362 (14·8%)	4699 (9·6%)	1·54 (1·39–1·70)
**Any mental health diagnosis**			
Before	1522 (62·0%)	14 455 (29·6%)	2·10 (2·03–2·17)
Subsequent	819 (33·4%)	7473 (15·3%)	2·18 (2·06–2·32)

Data are n (%), unless otherwise specified.

A total of 908 (37·0%) deaths occurred in the self-harm cohort compared with 12 683 (25·9%) deaths in the comparison cohort, of which 54 (5·9%) were unnatural deaths and 36 (4·0%) were suicides in the self-harm cohort versus 275 (2·2%) unnatural deaths and 12 (<0·1%) suicides in the comparison cohort (appendix). Mortality risk was found to change according to length of follow up, with markedly greater HRs observed for the first follow-up year (figure). Compared with their peers who had not harmed themselves, adults in the self-harm cohort were an estimated 20 times more likely to die unnaturally during the first year (IMD-adjusted HR 19·65 [95% CI 11·69–33·05]) after a self-harm episode and 3–4 times more likely to die unnaturally in subsequent years (3·41 [2·17–5·35; figure). The risk of death by suicide markedly increased after self-harm over the entire 13 years of follow up (IMD-adjusted HR 145·43 [95% CI 53·91–392·29]; appendix). Cumulative incidence plots and percentage values show that the self-harm cohort have markedly elevated risks for dying by any unnatural cause, and by suicide in particular, compared with the matched comparison cohort (appendix).

**Figure:**
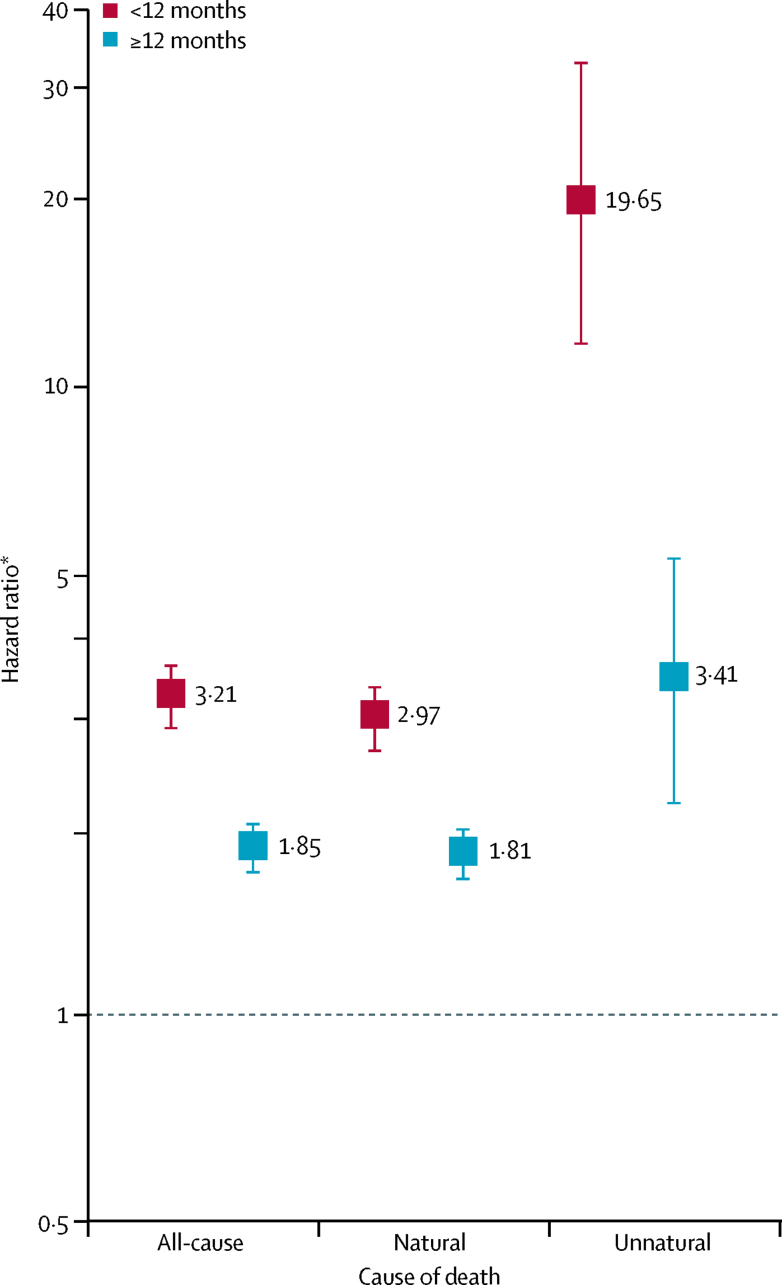
All-cause and cause-specific mortality risk during first 12 months and subsequent years of follow-up for the self-harm cohort versus the comparison cohort Comparison cohort matched with self-harm cohort for age, sex, and general practice using a log scale. *Adjusted for practice-level socioeconomic status.

## Discussion

This cohort study used CPRD, one of the world's largest electronic primary health-care datasets, and found that referral rates to mental health services were low, with women more likely to be referred than men, and likelihood of referral from general practices in more socially deprived localities was lower than from less deprived areas. Prescription rate was high for psychotropics within 12 months of the self-harm episode. 12% of the self-harm cohort were prescribed TCAs. Mental illness diagnosis was more than twice as prevalent with a higher level of multimorbidity among the self-harm cohort compared with the comparison cohort. We found that older adults (65 years and older) after self-harm were at increased risk of dying unnaturally, particularly of suicide, compared with individuals without a history of self-harm.

This is the first study to focus on self-harm exclusively among an older aged population from a primary care perspective. We report for the first time the clinical management in primary care during the first year after self-harm and found a low rate of referral to psychiatric services. NICE guidelines for short-term management of adults after self-harm^17^ recommend assessment by a mental health practitioner because of the more frequent suicidal intent among older people. In a previous study^4^ of adults aged 65 year and older, after contact at accident and emergency hospital departments in three English cities, 90% had an aftercare referral: 36% to outpatient care, 36% to a primary care GP, and 19% to inpatient care. In a similar study^6^ done in three hospitals in Manchester, UK, comparing middle-aged adults to older adults aged 55 years and older, the younger age group were more likely to be discharged to primary care than the older adults, and a greater number of older individuals were admitted to psychiatric hospital after a psychiatric assessment. However, 43% were discharged to primary care.

The NICE guidance for the long-term management of self-harm (CG133)^26^ and outlined in a Do Not Do Recommendation^27^ states, “When prescribing drugs for associated mental health conditions to people who self-harm, take into account the toxicity of the prescribed drugs in overdose…In particular, do not use tricyclic antidepressants, such as dosulepin, because they are more toxic”.

We report a high proportion of TCA prescribing after self-harm; TCAs are known to be fatally toxic in overdose and some TCAs more harmful than others.^28^ In a US observational study^29^ of adults attending accident and emergency departments for drug overdose between 2004 and 2007, the proportion of older adults using TCAs was significantly higher than in adults younger than 55 years. The authors suggested that the older age group were using TCAs before guideline changes regarding high toxicity in overdose or that TCAs might be prescribed for other conditions, such as neuropathic pain.

Few studies report mental health diagnoses before and after self-harm in older adults, with most based on psychological autopsy and suicide or investigating risk factors. In a retrospective cohort^30^ that used linked English hospital and mortality data, risk of self-harm was 15 times greater for adults aged 65 years and older diagnosed with depression, 14 times greater for those with bipolar disorder, 8 times greater for those with anxiety and neurotic disorders, and 6 times greater for those with a schizophrenia diagnosis than for those of a comparison cohort of day cases and inpatients without the psychiatric conditions. We report greater prevalence across all categories of mental illness investigated in the self-harm cohort. In an Australian population-based cohort^9^ of hospital admissions among older adults aged 50 years and older, 6·0% of those presenting with a self-harm injury had three or more comorbidities from the Charlson comorbidity index compared with 3·6% in the comparison cohort of individuals who were injured by some mechanism other than self-harm. Older individuals reportedly perceive physical health as one of the most common precipitants of self-harm.^5^, ^6^ Previous findings from a Danish study^11^ of adults aged 65 years and older, during a 20-year period from 1990 and 2009, found physical illnesses were important predictors of suicide and the association of physical illness with suicidal behaviour was acknowledged in a more recent systematic review.^14^

Within 12 months of initial self-harm, 14·4% of older adults had a further self-harm episode, which is similar to findings reported from a hospital-based study^4^ of adults aged 60 years and older (12·8%) attending five hospital emergency departments in England between 2000 and 2007. Among individuals in the same age range attending a hospital in Oxford, UK, during 1978–97, 8·2**%** had a repeat episode after deliberate self-harm.^5^ Although suicide cases were rare in our cohort from a primary care setting, the suicide risk was markedly elevated at 145 times higher than expected compared with 67 times higher based on a hospital setting.^4^

The higher rate of repetition found in our cohort and the markedly increased risk of suicide and unnatural death, particularly within 12 months of a known self-harm episode, identify a high-risk group of older individuals within primary care. Additionally, with older aged adults showing greater suicidal intent inferred from previous studies,^5^, ^6^, ^7^ this work should alert policy makers and primary health-care professionals to progress towards implementing preventive measures among older adults who consult with a GP. Primary health-care professionals have the opportunity to intervene since older adults are reported to consult more frequently than younger adults because of their complex needs and potentially higher levels of psychiatric and physical multimorbidity. Since drug ingestion is one of the main methods of self-harm, we also highlight the increasing urgency for the consideration of alternative medication and avoidance of TCAs in the older age group, both in mental health and pain management within primary care, and recommend maintaining frequent medication reviews after self-harm.

The low referral rates we report and the high rates of referral back to primary care after hospital admission from previous hospital-based studies, emphasise the importance of a GP's role to ensure individuals are assessed and monitored effectively after self-harming behaviour. Additionally, the inequity in referral across differing socioeconomic status highlights an important target for improvement across the health-care system.

As highlighted by a systematic review^31^ on interventions for reducing suicidal ideation among older adults, most studies were of low quality with the exception of two primary care-based, randomised controlled trials. PROSPECT, as part of a collaborative trial,^32^ investigated supportive care in depression of older people with the use of a combined intervention. This intervention involved improving the physicians' knowledge of geriatric depression, a major risk factor for suicide, in primary care and treatment management through first-line SSRI use. Suicidal ideation significantly declined in the intervention group compared with usual care.^32^ Promising non-pharmalogical interventions include an adapted interpersonal psychotherapy in older people to reduce suicidal ideation.^33^ The intervention was based on identifying and clarifying factors contributing to the individual's psychological distress, enhancing social connections, engaging in physical and social activities, and identifying negative interpersonal interactions. A significant reduction in suicidal ideation and depression symptom severity and significant increases in participant social adjustment and social interactions were found.

The present study did, however, have some limitations. First, the coding recorded within the CPRD and HES datasets is reliant on the clinicians who assign and enter the codes. Second, some hospital-treated cases of self-harm will not have been reported to an individual's GP and, therefore, will not have been captured in CPRD.^34^ As recommended by the authors, we linked the CPRD records to national mortality records to enable analysis of cause-specific mortality with complete ascertainment. Finally, studies investigating suicide tend to underestimate because coroners might be reluctant to return a verdict of suicide more frequently in unnatural deaths of older people who might have strong religious affiliations, and levels of stigma surrounding suicide among this age group might cause additional distress to their families. To address this issue, we included open verdicts in this regard, which is accepted practice in the UK.^35^

This novel primary-care-based study with linkage to hospital admissions and mortality records furthers understanding of self-harm among older aged adults. We have identified a group at high risk of premature unnatural death and identified areas of improvement for clinical management of older-aged adults in primary care. Health-care professionals should take the opportunity to consider the risk of self-harm when an older person consults with other health problems, especially when major physical illnesses and psychopathology are both present, to reduce the risk of an escalation in self-harming behaviour and associated mortality.

For more on the **code lists** see www.clinicalcodes.org

For the **Clinical Practice Research Datalink** see www.crpd.com/researcher

For the **UK Health and Social Care Information Centre** see www.hscic.gov.uk/hesdata

For the **UK Office for National Statistics** see www.ons.giv.uk/ons/index

## Data sharing
