# Supplementary material for: Self-harm in a primary care cohort of older people: incidence, clinical management, and risk of suicide and other causes of death
Source: Lancet Psychiatry. 2018 Nov;5(11):905–12. doi: 10.1016/S2215-0366(18)30348-1 (PMC6203699; doi:10.1016/S2215-0366(18)30348-1)
Supplement: Supplementary appendix [file mmc1.pdf]

THE LANCET
Psychiatry

Supplementary appendix

This appendix formed part of the original submission and has been peer reviewed. We post it as supplied by the authors.

Supplement to: Morgan C, Webb R, Carr M, et al. Self-harm in a primary care cohort of older people: incidence, clinical management and risk of suicide and other causes of death. *Lancet Psychiatry* 2018; published online Oct 15. http://dx.doi.org/10.1016/S2215-0366(18)30348-1.

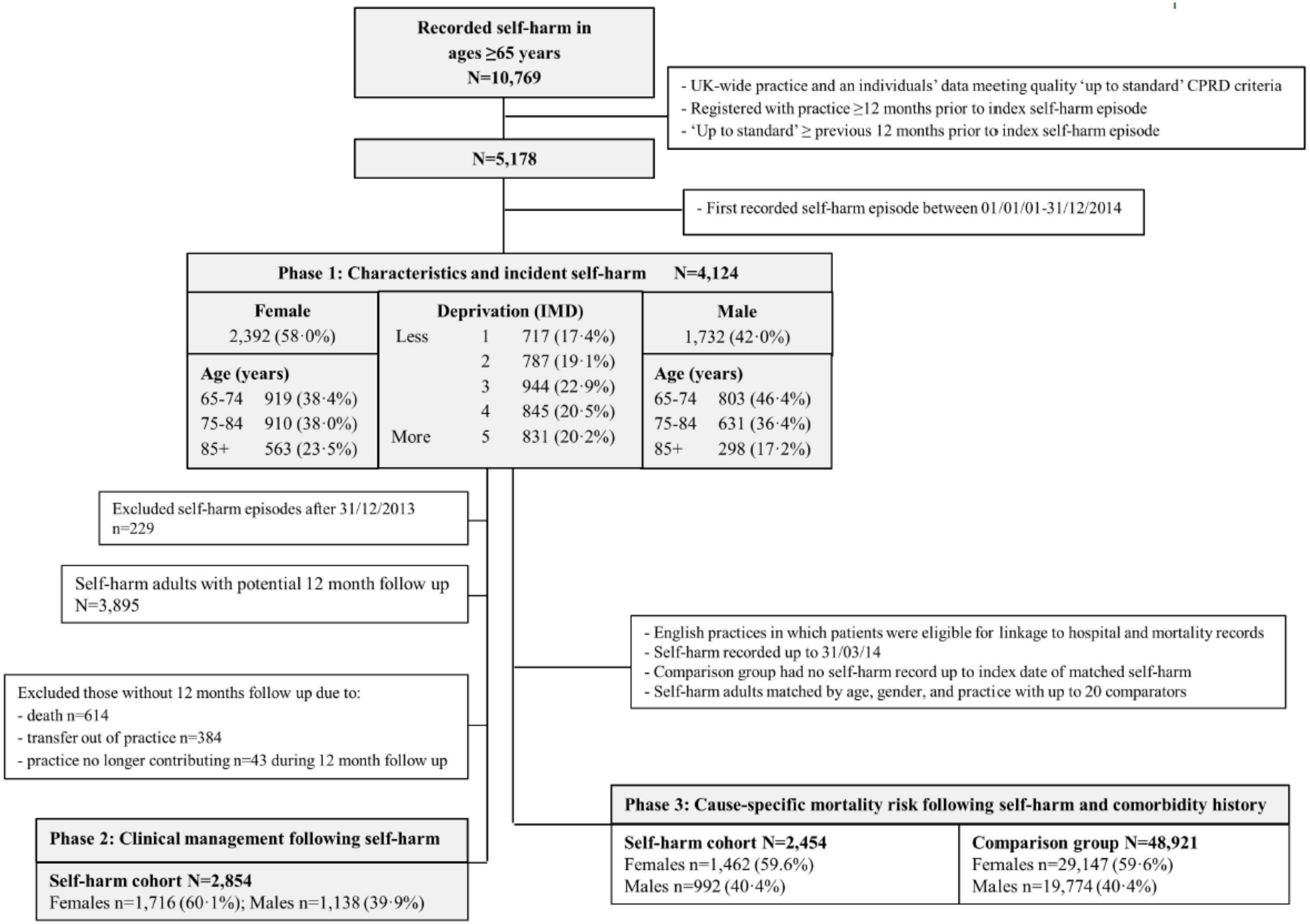

Figure S1: Summary of the derivation of each cohort for the three analytical phases

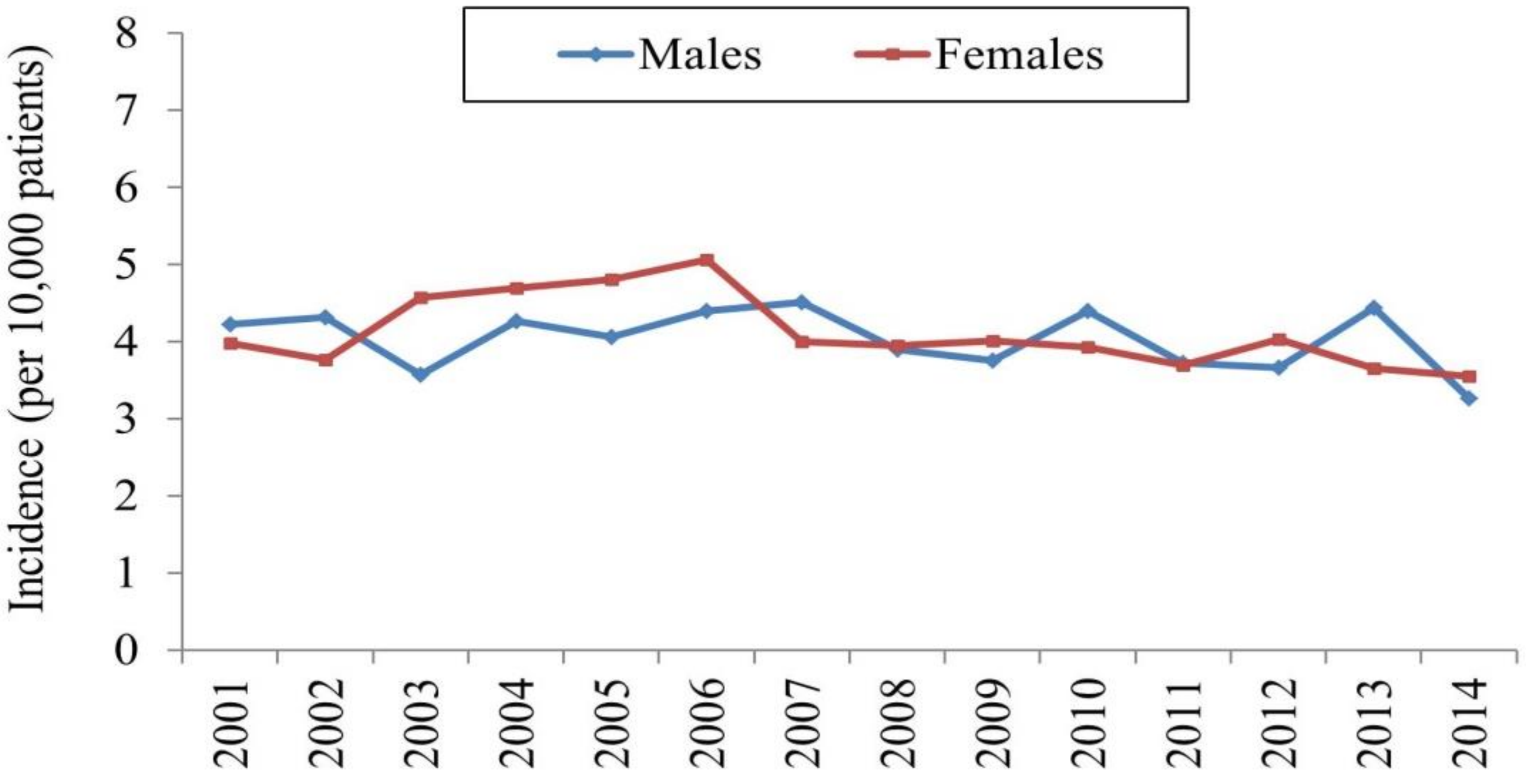

Figure S2: Temporal trends in standardised annual incidence rates stratified by gender

Co-morbid condition	Timing of illness to study entry	Self-harm (N=2,454) n (%)	Comparison (N=48,921) n (%)	Prevalence Rate Ratio (95% CI)
**Cancer**	- Prior to	445 (18·1)	7,100 (14·5)	1·25 (1·15, 1·36)
	- Subsequent	241 (9·8)	5,640 (11·5)	0·85 (0·75, 0·96)
**Cerebrovascular disease**	- Prior to	394 (16·1)	5,044 (10·3)	1·56 (1·42, 1·71)
	- Subsequent	332 (13·5)	4,558 (9·3)	1·45 (1·31, 1·61)
**Chronic pulmonary disease**	- Prior to	645 (26·3)	9,587 (19·6)	1·34 (1·25, 1·44)
	- Subsequent	169 (6·9)	3,011 (6·2)	1·12 (0·96, 1·30)
**Congestive heart disease**	- Prior to	328 (18·3)	3,568 (7·3)	1·83 (1·65, 2·04)
	- Subsequent	244 (9·9)	4,150 (8·5)	1·17 (1·04, 1·32)
**Diabetes**	- Prior to	397 (16·2)	6,075 (12·4)	1·30 (1·19, 1·43)
	- Subsequent	88 (3·6)	2,355 (4·8)	0·74 (0·60, 0·92)
**Diabetes with complications**	- Prior to	112 (4·6)	1434 (2·9)	1·56 (1·29, 1·88)
	- Subsequent	50 (2·0)	1,073 (2·2)	0·93 (0·70, 1·23)
**Hemiplegia**	- Prior to	59 (2·4)	574 (1·2)	2·05 (1·57, 2·67)
	- Subsequent	54 (2·2)	614 (1·3)	1·75 (1·33, 2·31)
**Metastatic tumour**	- Prior to	66 (2·7)	605 (1·2)	2·17 (1·69, 2·79)
	- Subsequent	118 (4·8)	2,049 (4·2)	1·15 (0·96, 1·38)
**Mild/moderate liver disease**	- Prior to	36 (1·5)	297 (0·6)	2·42 (1·71, 3·41)
	- Subsequent	23 (0·9)	294 (0·6)	1·56 (1·02, 2·38)
**Myocardial infarction**	- Prior to	238 (9·7)	3,408 (7·0)	1·39 (1·23, 1·58)
	- Subsequent	105 (4·3)	1,951 (4·0)	1·07 (0·89, 1·30)
**Peptic ulcer disease**	- Prior to	238 (9·7)	3,455 (7·1)	1·37 (1·21, 1·56)
	- Subsequent	70 (2·9)	1,340 (2·7)	1·00 (0·82, 1·32)
**Peripheral vascular disease**	- Prior to	213 (8·7)	3·017 (6·2)	1·41 (1·23, 1·61)
	- Subsequent	121 (4·9)	2,028 (4·2)	1·19 (0·99, 1·42)
**Renal disease**	- Prior to	430 (17·5)	6,911 (14·1)	1·24 (1·14, 1·36)
	- Subsequent	426 (17·4)	8,906 (18·2)	0·95 (0·87, 1·04)
**Rheumatological disease**	- Prior to	214 (8·7)	3,151 (6·4)	1·35 (1·19, 1·55)
	- Subsequent	59 (2·4)	1,337 (2·7)	0·88 (0·68, 1·14)
**Any physical health diagnosis**	- Prior to	1,760 (71·7)	29,341 (60·0)	1·20 (1·17, 1·23)
	- Subsequent	1,214 (49·5)	22,729 (46·5)	1·01 (1·02, 1·11)
**Charlson Index scores**		**Self-harm (N=2,454) n (%)**	**Comparison (N=48,921) n (%)**	**Prevalence Rate Ratio (95% CI)**
**0-2**		848 (24·54)	23,410 (47·85)	0·72 (0·68, 0·76)
**3-5**		837 (34·11)	15,016 (30·69)	1·11 (1·05, 1·18)
**6-8**		533 (21·07)	7551 (15·4)	1·41 (1·30, 1·52)
**9+**		236 (9·62)	2944 (6·01)	1·60 (1·41, 1·81)

Table S1: Prevalence of specific comorbid physical health conditions and distribution of Charlson Index scores in self-harm and comparison cohorts

Death by	Risk period	Self-harm cohort (N=2,454)		Comparison cohort (N=48,921)		Hazard ratio (95% CI)	
	Months	No. of deaths	Rate/1000PYs	No. of deaths	Rate/1000PYs	Unadjusted	Adjusted†
**All causes**	≤ 12	330	164·31	2,415	53·38	3·23 (2·88, 6·68)	3·21 (2·85, 3·61)
	> 12	578	97·36	10,268	57·27	1·86 (1·71, 2·03)	1·85 (1·70, 2·02)
**Natural causes**	≤ 12	301	149·87	2,374	52·47	2·99 (2·65, 3·38)	2·97 (2·63, 3·35)
	> 12	553	93·15	10,034	55·97	1·83 (1·67, 2·00)	1·81 (1·66, 1·98)
**Unnatural causes**	≤ 12	29	14·44	41	0·91	19·74 (11·74, 33·20)	19·65 (11·69, 33·05)
	> 12	25	4·21	234	1·31	3·41 (2·17, 5·35)	3·41 (2·17, 5·35)
**Suicide‡**	Full period	36	4·53	12	0·05	131·28 (51·45,335·01)	145·43 (53·91,392·29)

† Adjusted by Index of Multiple Deprivation quintile

‡ Analysis period not shown for ≤12 months and >12 months due to cell count <5 cases

PYs: Person years

Table S2: All-cause and cause-specific mortality risk during first 12 months and subsequent years of follow-up for the self-harm cohort versus the age-, gender- and practice-matched comparison cohort

a) All-cause of death

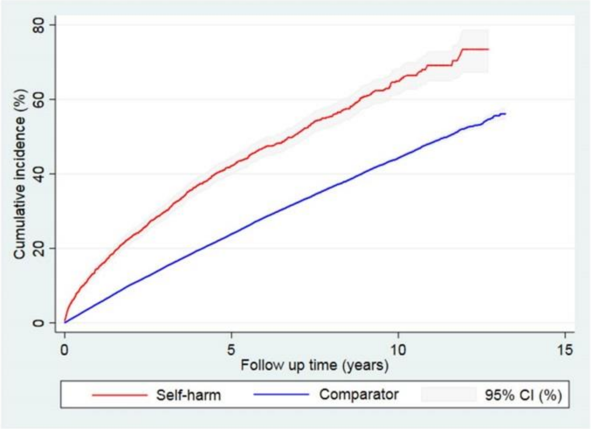

	All-Cause - Cumulative incidence % (95% CI)		
**Cohort**	**At 1 year**	**At 5 years**	**At 10 years**
Self-harm	14·63 (13·21, 16·11)	42·20 (39·73,44·64)	64·95 (61·25, 68·38)
Comparison	5·23 (5·03, 5·43)	23·87 (23·42, 24·31)	44·2 (43·54, 44·98)

b) Natural cause of death

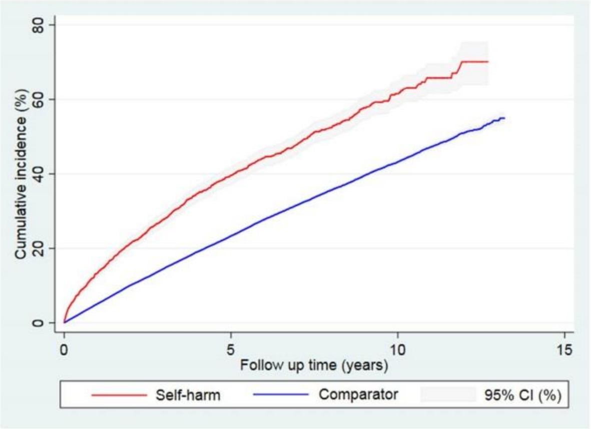

	Natural death - Cumulative incidence† % (95% CI)		
**Cohort**	**At 1 year**	**At 5 years**	**At 10 years**
Self-harm	13·35 (11·99, 14·79)	39·62 (37·19, 42·04)	61·57 (57·90, 65·02)
Comparison	5·14 (4·94, 5·34)	23·37 (22·92, 23·81)	43·23 (42·51, 43·95)

†Adjusted for competing risk of unnatural death

c) Unnatural cause of death

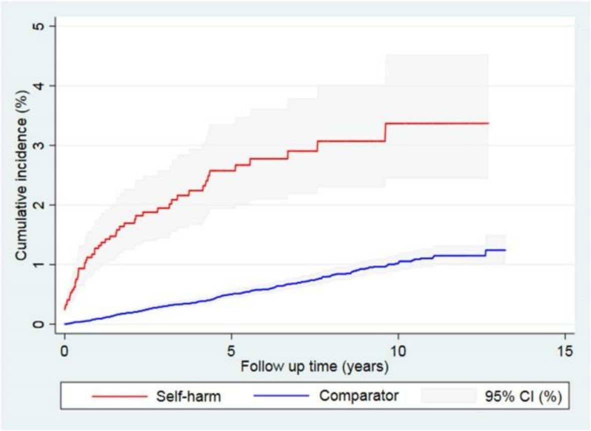

	Unnatural death - Cumulative incidence† % (95% CI)		
**Cohort**	**At 1 year**	**At 5 years**	**At 10 years**
Self-harm	1·27 (0·90, 1·75)	2·57 (1·93, 3·36)	3·37 (2·44, 4·53)
Comparison	0·088 (0·065, 0·12)	0·50 (0·43, 0·58)	1·03 (0·90, 1·18)

†Adjusted for competing risk of natural death

d) Death by suicide

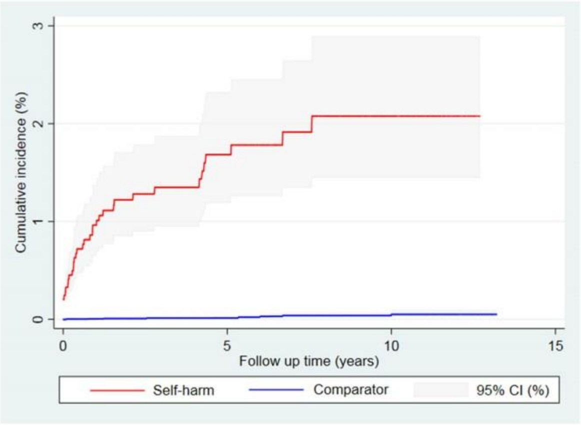

	Suicide - Cumulative incidence† % (95% CI)		
**Cohort**	**At 1 year**	**At 5 years**	**At 10 years**
Self-harm	1·27 (0·90, 1·75)	1·68 (1·19, 2·32)	2·08 (1·44, 2·90)
Comparison	0·0043 (0·00097, 0·016)	0·015 (0·0065, 0·033)	0·052 (0·025, 0·099)

†Adjusted for competing risk of dying by any other cause

Figure S3: Cumulative incidence of death due to all causes and specific causes in the self-harm and comparison cohort
